# Stromal Vascular Fraction Transplantation as an Alternative Therapy for Ischemic Heart Failure: Anti-inflammatory Role

**DOI:** 10.1186/1749-8090-6-43

**Published:** 2011-03-31

**Authors:** Goditha U Premaratne, Li-Ping Ma, Masatoshi Fujita, Xue Lin, Entela Bollano, Michael Fu

**Affiliations:** 1 Wallenberg Laboratory for Cardiovascular Research, Sahlgrenska University Hospital, University of Gothenburg, Gothenburg, Sweden; 2 Department of Cardiology, Shanghai Second Military Medical University, Shanghai, PR China; 3 Department of Human Health Sciences, Graduate School of Medicine, Kyoto University, Kyoto, Japan

## Abstract

**Background:**

The aims of this study were: (1) to show the feasibility of using adipose-derived stromal vascular fraction (SVF) as an alternative to bone marrow mono nuclear cell (BM-MNC) for cell transplantation into chronic ischemic myocardium; and (2) to explore underlying mechanisms with focus on anti-inflammation role of engrafted SVF and BM-MNC post chronic myocardial infarction (MI) against left ventricular (LV) remodelling and cardiac dysfunction.

**Methods:**

Four weeks after left anterior descending coronary artery ligation, 32 Male Lewis rats with moderate MI were divided into 3 groups. SVF group (n = 12) had SVF cell transplantation (6 × 10^6 ^cells). BM-MNC group (n = 12) received BM-MNCs (6 × 10^6^) and the control (n = 10) had culture medium. At 4 weeks, after the final echocardiography, histological sections were stained with Styrus red and immunohistochemical staining was performed for α-smooth muscle actin, von Willebrand factor, CD3, CD8 and CD20.

**Results:**

At 4 weeks, in SVF and BM-MNC groups, LV diastolic dimension and LV systolic dimension were smaller and fractional shortening was increased in echocardiography, compared to control group. Histology revealed highest vascular density, CD3+ and CD20+ cells in SVF transplanted group. SVF transplantation decreased myocardial mRNA expression of inflammatory cytokines TNF-α, IL-6, MMP-1, TIMP-1 and inhibited collagen deposition.

**Conclusions:**

Transplantation of adipose derived SVF cells might be a useful therapeutic option for angiogenesis in chronic ischemic heart disease. Anti-inflammation role for SVF and BM transplantation might partly benefit for the cardioprotective effect for chronic ischemic myocardium.

## Background

Cell transplantation is an effective treatment of repairing ischemically damaged hearts [1,2]. The use of stem cells emerged as a reasonable alternative treatment and two general types of stem cells are being used for this aspect [3,4]. Although theoretically highly applicable, there are some potential limitations of cell regulation and ethical considerations for the practical use of embryonic stem cells [4]. Bone marrow mono nuclear cells (BM-MNCs) have been the most commonly used stem cells for ischemic myocardium, probably due to the availability of multipotential progenitor cells. Mesenchymal stem cells (MSCs) are multipotent adult stem cells that reside within the bone marrow microenvironment. Although mesenchymal stem cells derived from bone marrow have been used experimentally [2,3] and clinically [5,6], bone marrow aspiration is very painful and sometimes requires the use of general or spinal anaesthesia. Therefore, an autologous pluripotent mesenchymal stem cell source that allows harvesting in large numbers with minimal discomfort would be ideal for transplantation. Adipose tissue is derived from embryonic mesoderm and contains a heterogeneous stromal cell population that can be easily harvested from the patients by a simple, minimally invasive method, and they can be easily cultured. Several studies have demonstrated the presence of uncommitted MSCs within the adipose tissue of animals and humans [7,8], that have the ability to regenerate damaged organs. In addition, it has been reported that MSCs derived from adipose tissue are multipotent cells that can differentiate into cardiomyocytes [9,10] and vascular endothelial cells [11,12]. Therefore, adipose-derived stromal vascular fraction (SVF) emerging as a better option to replace bone marrow for implantation into ischemic myocardium using easy and non-invasive procedures.

Although, the effects of adipose-derived SVF transplantation into ischemic myocardium have been recently reported [13], underline mechanisms of adipose-derived cells transplanted into chronic ischemic myocardium have not yet been established. Therefore, this study investigated the therapeutic efficacy of adipose-derived SVF cells or freshly isolated BM-MNCs in a rat model of chronic myocardial infarction and the anti-inflammatory role of engrafted SVF and BM-MNC in post chronic myocardial infarction.

## Methods

### Experimental Animals

Adult male syngeneic Lewis rats weighing 250-290 g were used as recipients and donors in this study. All experimental procedures were approved by the regional Animal Ethic Committee of Gothenburg University, Gothenburg, Sweden and conducted in accordance with the *Guide for the Care and Use of Laboratory Animals *published by the US National Institute of Health (NIH publication no.85-23, revised 1996).

### Stromal Vascular Fraction (SVF) Isolation

Stromal vascular fraction was isolated as Zuk et al. described with some modifications [14]. Adipose tissue was obtained from the inguinal region of syngeneic Lewis rats under sterile conditions, kept in the tissue culture media on ice, washed extensively with phosphate-buffered saline (PBS) to remove contaminating blood cells, dissected from vessels and minced with scissors. Minced adipose tissue was enzymatically digested using PBS containing 2% BSA and collagenase (0.2%) at 37°C for 45 minutes; the enzyme reaction was inactivated by the addition of DMEM/Ham's F-12 (PAA Laboratories GmbH, Haidmannweg, Pasching, Austria) containing 10% newborn calf serum (NCS) and centrifuged at 800 g for 10 minutes to obtain a high density SVF pellet. The pellet was resuspended in 160 mM NH_4_Cl for 15 minutes at room temperature to lyse red blood cells, added equal volume of DMEM/Ham's F-12 containing 10% NCS, centrifuged at 800 g for 10 minutes. The cell suspension was filtered through a 100 μm nylon mesh to remove undispersed tissue elements and plated (30 000 cells/cm^2^) in DMEM-F12 containing 10% NCS. Six hours after incubation, the plates were washed extensively with PBS to remove residual non-adherent red blood cells. Cells were labeled with a fluorescent dye using PKH26 (PKH26 Red Fluorescent Cell Linker Mini Kit, for General Cell Membrane Labeling, SIGMA-ALDRICH Inc.) [15]. Cells were suspended at a concentration of 6 × 10^7^/mL in 0.1 mL culture medium (without serum) for transplantation.

### Bone marrow mononuclear cell (BM-MNC) Isolation

BMCs were harvested from 8-week syngeneic Lewis rats by flushing the femurs and tibias with PBS supplemented with 2% fetal bovine serum. To isolate mononuclear cells, the gradient centrifugation method with Percoll was used [16]. After the cells were washed in PBS for 3 times, labeled with a fluorescent dye using PKH26, before suspended in 0.1 mL of culture medium (without serum) at a concentration of 6 × 10^7^/mL cells for transplantation.

### Chronic myocardial infarction model

The animal model, which was employed in this study, has been described previously [17]. Male Lewis rats weighing 250-290 g were anesthetized with isoflurane, orally intubated into the trachea and anesthesia was maintained with 1.5% to 2.5% isoflurane during the ligation procedure. They underwent a left lateral thoracotomy, the left anterior descending coronary (LAD) artery was ligated with a 6-0 polypropylene suture (Ethicon, Inc, Somerville, NJ). As a result, ST-segment elevation on electrocardiogram and color changes in the left ventricular (LV) myocardium were observed in all rats.

### Experimental Groups

Four weeks after LAD ligation, infarction size was evaluated by echocardiography and rats with moderate-sized infarction (infarct size, 20% to 40%) were randomized into 3 groups. In SVF Group (n = 11), SVF 6 million cells suspended in culture medium were subepicardially implanted at 2 points of the border zone. In BM-MNC group (n = 11), 6 × 10^6 ^bone marrow mono nuclear cells were transplanted. Control group (n = 10) received culture medium injection. Fresh DMEM culture medium without serum was used for all the injections. Thus, all the 32 rats had repeat thoracotomy for the myocardial injection.

### Echocardiography

Rats were anesthetized with isoflurane. Left ventricular function was studied just before transplantation and followed-up 2 and 4 weeks later, by echocardiography with an ultrasound machine (HDI 5000 ultrasound system, ATL, Philip Medical System, Best, Netherlands) equipped with a 12 MHz phased-array transducer. A two-dimensional short-axis view of the LV was obtained at the level of the papillary muscles, M-mode images were recorded at the same plane and LV end-diastolic dimension (EDD) and end-systolic dimension (ESD) were measured. In addition, the percentage of fractional shortening (FS) was calculated. All measurements were performed in a blind fashion according to the American Society for Echocardiology, and averaged over 3 consecutive cardiac cycles.

### Histology

After echocardiographic assessment, all rats were sacrificed, hearts from each group were cryo-embedded and the whole left ventricle was sectioned in 4 μm thickness along the short axis. They were microscopically examined with the use of fluorescence microscopy for PKH26 dye. The sections were stained for hematoxylin and eosin. Immunohistochemistry was performed for α-sarcomeric actin, von Willebrand factor (Dako Cytomation Inc, Glostrup, Denmark), Interleukin-6 (IL-6) (Abcam plc., UK), CD3 (Santa Cruz Biotechnology, Inc., Europe), CD8 (Santa Cruz Biotechnology, Inc., Europe) and CD20 (Santa Cruz Biotechnology, Inc., Europe).

In addition, Sirus red staining was performed to examine the fibrosis percentage in the infarct area with an image analysis software (Scion Image Beta 4.02 Win, Photoshop 6.0, San Jose, CA, USA).

### Analysis of Vascular Density

The number of vessels was counted in each heart using immunohistochemistry for von Willebrand factor [15]. The vessels per 1 mm^2 ^in the peri-infarct zone were counted in 5 randomly chosen fields per slide in a blinded manner in 5 sections from each heart and averaged for statistical analysis.

### Analysis of Fibrotic Area

The percentage of fibrotic area in the infarct and peri-infarct zone was calculated in each heart using the image analysis software (Scion Image Beta 4.02 Win, Scion Corporation) in a representative preparation for Sirius Red staining, with the red areas regarded as fibrotic. The percentage of fibrotic area was analyzed in 5 randomly chosen fields per slide in the infarct and peri-infarct zone in a blinded manner in 5 sections from each heart and averaged for statistical analysis.

### Isolation of RNA and real time RT-PCR

Total RNA was isolated from left ventricular myocardium using SV total RNA Isolation System (Promega, Madison, WI, USA) according to the manufacturer's recommendations. Reverse transcriptase reaction using TaqMan High capacity cDNA Archive Kit (Applied Biosystems, Foster City, CA, USA) was performed for cDNA synthesis. The cycling parameters were 25°C for 10 minutes and 37°C for 2 hours.

Real time RT-PCR analyses were used to determine mRNA expressions of tumor necrosis factor alpha (TNFα), Interleukin-6 (IL-6), tissue inhibitor of matrix metalloproteinase-1 (TIMP-1), matrix metalloproteinase-1 (MMP-1), brain natriuretic peptide (BNP) and vascular endothelial growth factor (VEGF), and were performed with TaqMan Assay-on-Demand on ABI 7700 sequence Detection System (ABI), according to the manufacturer's recommendations. The expression data were normalized to an endogenous control, β-glucuronidase (Gus B). The reactions for TNFα, IL-6, TIMP-1, MMP-1, BNP and VEGF were analyzed in duplicates and the relative expression levels were calculated according to the standard curve method. The logarithm of the RNA concentration was calculated from standard curves. The expression was determined as the ratio of the RNA_target_/RNA_GusB_.

### Positive cells for CD3, CD8 and CD 20

Immunohistochemical staining was performed on left ventricular sections using anti-CD3, anti-CD8 and anti-CD20. The diffusely scattered positive cells were counted in each image. The visual field area of the x20 objective of the light microscope used; the positive cells in four consecutive fields of representative areas were counted in 5 sections from each heart and averaged for statistical analysis.

### Quantification of IL6 positive immunohistochemical staining

For quantification of IL6 positive area, the immunopositive components from the images from each section were dissected using the property of color recognition of BioPix iQ 2.1.6 softaware. The percentage of IL6 positive area was analyzed in 4 randomly chosen fields per slide in the infarct area in a blinded manner in 5 sections from each heart and averaged for statistical analysis.

### Data Analysis

All data are expressed as the mean ± SEM. Comparisons of echocardiographic data among the groups were performed by 2 way repeated measures analysis of variance (ANOVA) including time, group, and group-by-time interaction terms. If significance was recognized for the group effect or the group-by-time interaction, post hoc comparisons among the groups or among the groups at each time point were performed, and if significance was found for the time effect or the group-by-time interaction, post hoc comparisons among the time points in each group were made, when appropriate, using Fisher's protected least significant difference method. Comparisons of vascular density data, fibrosis and mRNA expressions among the groups were conducted by one-way factorial ANOVA. All statistical analyses were performed with using computer software (Stat View for Windows version 5.0, SAS Institute Inc, Cary, NC, USA). A probability value < 0.05 was considered statistically significant.

## Results

### Mortality

The mortality rate due to coronary artery ligation was 20%. There was no intraoperative or postoperative death concerning treatment procedures.

### Echocardiography

Echocardiographic data are shown in Table 1. There were no differences among the 3 groups regarding pretreatment LVDd, LVDs and FS. Four weeks after each treatment, both LVDd and LVDs in the SVF and BM-MNC groups were significantly smaller than those in the control group (P < 0.05). The SVF and BM-MNC groups had better fractional shortening and ejection fraction than the control group.

**Table 1:** Echocardiographic data at pretreatment and 4 Weeks after cell or culture medium transplantation in 3 Groups

	SVF	BMMNC	Control
***Pre treatment***			
LVDd (cm)	0.92 ± 0.02	0.91 ± 0.02	0.92 ± 0.02
LVDs (cm)	0.68 ± 0.03	0.66 ± 0.03	0.68 ± 0.03
FS (%)	26.7 ± 1.6	28.5 ± 2	26.1 ± 2.6
EF (%)	57.0 ± 2.6	59.5 ± 3.1	55.2 ± 3.9
***After treatment***			
LVDd (cm)	0.88 ± 0.02*	0.93 ± 0.03*	1.02 ± 0.09
LVDs (cm)	0.60 ± 0.03*	0.65 ± 0.03*	0.78 ± 0.15
FS (%)	31.6 ± 2.6*	30.3 ± 1.7*	23.3 ± 1.1
EF (%)	63.8 ± 3.5*	62.5 ± 2.7*	51.2 ± 1.9

Values are shown as the mean ± SEM. LVDd, left ventricular end-diastolic dimension; LVDs, left ventricular end-systolic dimension; FS, fractional shortening; EF, ejection fraction. * *p *< 0.05 versus Control group.

### Cell transplants

**Figure 1:**
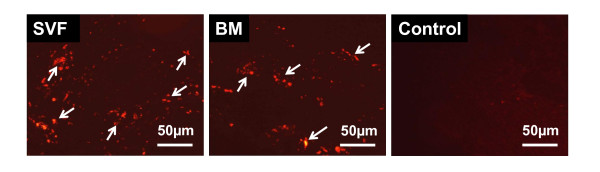
**Transplanted cells**. PKH26 labeled donor cells (red fluorescence, x200) in SVF and BM-MNC transplanted groups. Bars represent a distance of 50 μm.

PKH26 labelled transplanted cells were detected in host myocardium by their intense red fluorescence, 4 week after cell implantation. (Figure 1).

### Effects of cell therapy on vascular density

**Figure 2:**
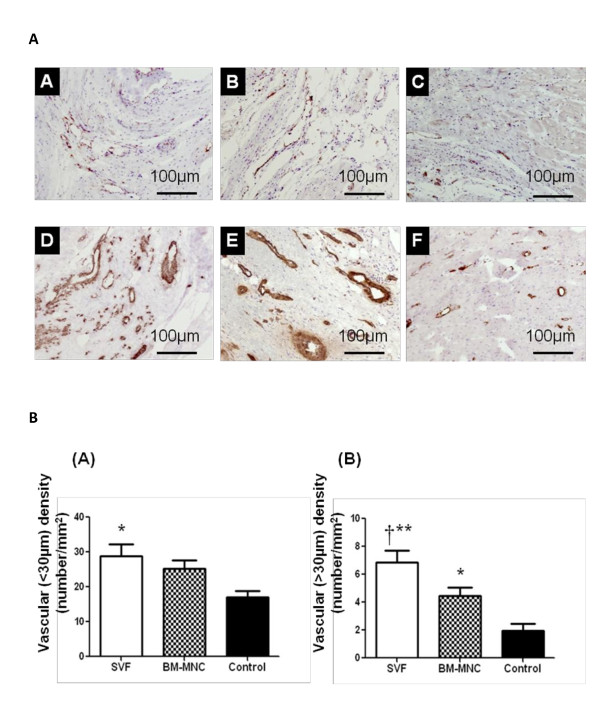
**Vascular density**. **2a **(A-C) Immunohistochemistry for von Willebrand factor (brown, x100). Representative pictures in the peri-MI area from SVF, BM-MNC and Control groups, respectively. (D-F) Immunohistochemistry with α-smooth muscle actin antibody (brown, x100). Representative pictures in the peri-MI area from SVF, BM-MNC and Control groups, respectively. Scale bars indicate distances of 100 μm. **2b **Graphs: the number of vessels (number/mm^2^) in the peri-MI area, micro-vessel density (density of vessels <30 μm in diameter) (A), and large-vessel density (density of vessels >30 μm in diameter) (B). Data are given as the mean ± SEM. *p < 0.05 vs. Control group, **p < 0.05 vs. BM-MNC group, **^†^**p < 0.001 vs. Control group.

Microscopic examination showed the following findings. There were many neovessels in and around the scar tissue 4 weeks after the injections of SVF and BM-MNC. Representative images are shown in Figure 2a. The vascular density of vessels larger than 30 μm in diameter in the peri-MI area was highest in the group with SVF (SVF, BM-MNC, Control: 6.88 ± 2.03, 4.45 ± 1.45 and 1.95 ± 1.19/mm2, respectively; p < 0.001). The vascular density in the groups with SVF and BM-MNC were significantly higher than the control group. Microvessel (<30μm) numbers were significantly lower in control rats than the SVF implanted group. (SVF, BM-MNC, Control: 28.78 ± 3.5, 25.17 ± 2. 54 and 17.11 ± 4.18/mm2, respectively; p < 0.05). Results of post hoc analysis were shown with symbols in Figure 2b.

### Fibrotic area inside the infarct and peri-infarct zone

**Figure 3:**
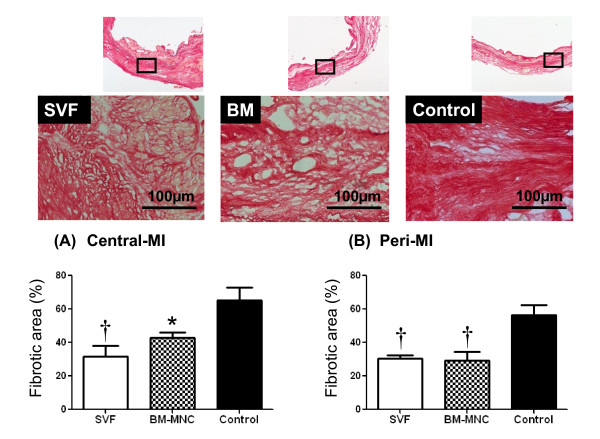
**Fibrotic area**. Representative pictures from groups SVF, BM-MNC and Control, respectively. Bars represent a distance of 100 μm. Graphs: Percentage of fibrotic area inside the infarct (A) and peri-infarct area (B). Data are given as the mean ± SEM. *p < 0.05 vs. Control group, **^†^**p < 0.01 vs. Control group.

The percentage of fibrotic area inside the infarct area was less in SVF and BM-MNC groups than that of control group (SVF, BM-MNC, Control: 31.84 ± 6.2, 42.88 ± 3.1 and 65.11 ± 7.86%, respectively; p < 0.01; Figure 3A). The percentage of fibrotic area inside the peri-infarct area was directionally similar to that of the infarct area. (SVF, BM-MNC, Control: 30.30 ± 2.35, 29.14 ± 5.5 and 56.39 ± 6.3%, respectively; p < 0.01; Figure 3B).

### SVF transplantation decreased gene expression of inflammatory cytokines TNFα and IL6

**Figure 4:**
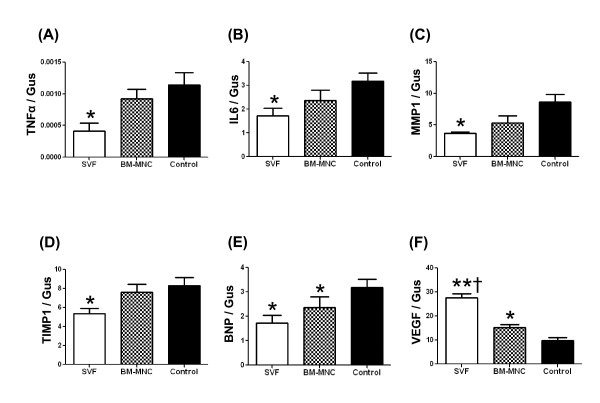
**Expression of mRNA**. Expression of mRNA levels of tumor necrosis factor α (A, TNFα); interleukin 6 (B, IL-6); matrix metalloproteinase 1 (C, MMP-1); tissue inhibitor of metalloproteinase 1 (D, TIMP-1), brain natriuretic peptide (E, BNP) and vascular endothelial growth factor (F, VEGF) in the left ventricular myocardium as measured by reverse transcription polymerase chain reaction in the rat left ventricular myocardium, 4 weeks after treatment. mRNA expressions were calculated via a standard curve and normalized to an endogen control. Data are given as the mean ± SEM. *p < 0.05 vs. Control group, **p < 0.01 vs. BM-MNC group, †p < 0.001 vs. Control group.

Expression of TNFα and IL6 mRNA was lower in the LV myocardium from the SVF group than the culture medium-injected control group following cell/culture medium treatment (P < 0.05; Figure 4A, and 4B). In the BM-MNC injected LV tissue, no significant differences were observed in TNFα or IL-6, mRNA levels, either with SVF or culture medium-injected LV myocardium. A high decrease in mRNA expression was noted in TNFα and IL-6 in the BM-MNC group rats compared with the control group, although these results did not reach statistical significance.

### SVF transplantation reduced MMP1 and TIMP1 gene expression

The mRNA analysis demonstrated decreased expression of MMP-1 and TIMP-1 in the SVF group as compared with the control group (P < 0.05; Figure 4C, and 4D). A high decrease in mRNA expression was noted in MMP-1 in the BM-MNC group rats compared with the control group, although these results did not reach statistical significance.

### BNP and VEGF mRNA expression

As shown in Figure 4E and 4F, the expression of BNP mRNA was lower and the expression of VEGF mRNA was higher in the LV myocardium from the SVF group compared with the culture medium-injected control group (P < 0.05), following 4 weeks treatment.

### Immunohistochemical studies for CD3, CD8 and CD 20

**Figure 5:**
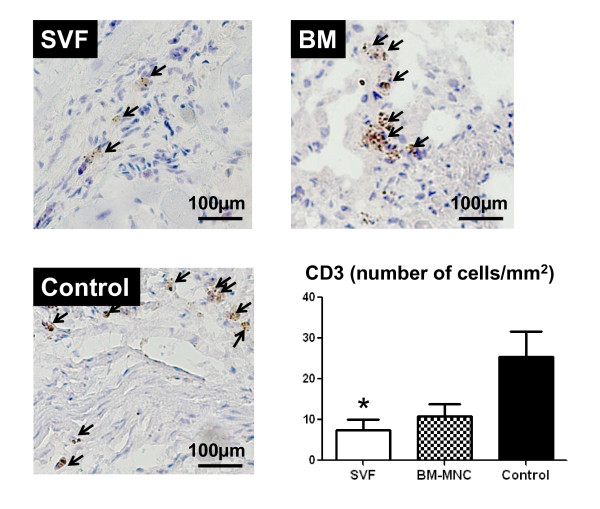
**Immunohistochemistry for CD3+ (T lymphocytes), (brown, × 100)**. Representative pictures in the infarct area from SVF, BM-MNC and Control groups, respectively. Bars represent a distance of 100μm. Graph: the number of CD3+ (number/mm^2^) in the infarct area. Data are given as the mean ± SEM. *p < 0.05 vs. Control group.

**Figure 6:**
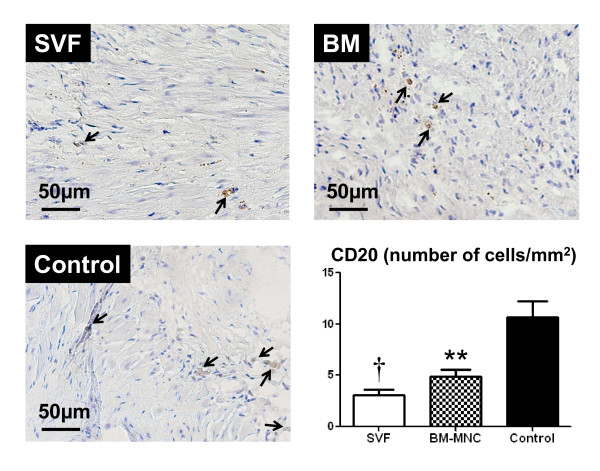
**Immunohistochemistry for CD20+ (B lymphocytes), (brown, × 100)**. Representative pictures in the infarct area from SVF, BM-MNC and Control groups, respectively. Bars represent a distance of 50μm. Graph: the number of CD20+ (number/mm^2^) in the infarct area. Data are given as the mean ± SEM. ^†^p < 0.001 vs. Control group, **p < 0.01 vs. Control group.

The mean number of cells positive for CD3 was decreased significantly in SVF transplanted rats compared to controls (p < 0.05; Figure 5). The mean number of CD20+ cells in the infarct was decreased significantly in SVF and BM transplanted rats compared to controls (p < 0.001, p < 0.01 respectively; Figure 6). In the cell transplanted groups, the number of CD8+ cells was not significantly different from the culture medium injected controls.

### Presence of IL-6 protein in the heart

**Figure 7:**
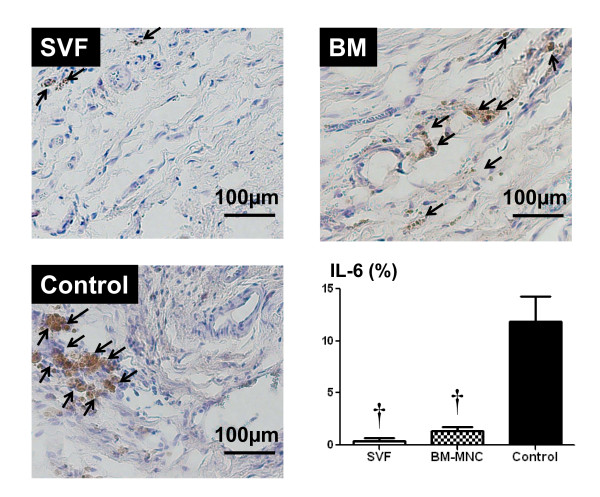
**Localization of IL-6 (brown) by immunohistochemical analysis in cell transplanted and control hearts. Magnification × 100**. Representative pictures in the infarct area from SVF, BM-MNC and Control groups, respectively. Bars represent a distance of 100μm. Graph: Percentage of IL-6 positive area inside the infarct. Data are given as the mean ± SEM. **^†^**p < 0.001 vs. Control group.

The percentage of area positive for IL-6 inside the LV myocardium 4 weeks after treatment was less in SVF and BM-MNC groups than that of control group (SVF, BM-MNC, Control: 0.38 ± 0.27, 1.33 ± 0.4 and 11.83 ± 2.41%, respectively; p < 0.001; Figure 7).

## Discussion

Cell therapy may be an alternative treatment for heart failure. The optimal cell for transplantation and the source of the cells to be isolated are important considerations. It has led to the investigations of different types of stem cell therapy for therapeutic angiogenesis. Several recent studies in animals [2,3] as well as humans [5,6] have repeatedly demonstrated that the transplantation of adult bone marrow derived cells can improve left ventricular function and inhibit adverse remodeling after myocardial infarction. The cardioprotective benefits may be mainly derived from the enhancement of neovascularization by BM cells, either by their ability to supply large amounts of angiogenic, anti-apoptotic and mitogenic factors [18] or by differentiating into vascular cells [11] and cardiomyocyte-like cells [12,19]. Unfortunately, the positive initial results of phase I/II studies remains highly controversial [20]. Moreover, bone marrow can only be obtained by bone marrow biopsy, a potentially painful procedure. Therefore, alternative source of stem cells or progenitors for therapeutic angiogenesis has been desired.

In this study, we focused on the protective effects of SVF transplantation compared to those of BM-MNC transplantation and the anti-inflammatory role of transplanted cells after implanted into a rat chronic myocardial infarction. Survived donor cells in host myocardium were clearly visualized with red fluorescence in SVF and BM-MNC implanted groups (Figure 1).

Major findings of the present study are summarized as follows. (1) Intramyocardial injection of SVF was more effective than that of BM-MNC or culture medium in enhancing neovascularization, inhibiting collagen deposition and reducing gene expression of inflammatory cytokines TNF-α, IL-6, TIMP-1 and BNP as well as inflammatory cells CD3, in rat chronic ischemic myocardium.; (2) Both the SVF and BM-MNC transplantation improved cardiac function, attenuated LV dilation, and thus prevented further myocardial remodelling.

Injection of SVF into ischemic myocardium was not associated with any side effects; specially, there were no casualties or arrhythmias due to cell implantation and there was no evidence of local infection. In this report, we demonstrated that direct intramyocardial injection of adipose derived SVF was more effective than BM-MNC or culture medium in enhancing neovascularization and improvement of LV function in chronic ischemic myocardium. By the ability of the other subpopulations of SVF and BM, including hematopoietic stem cells and mesenchymal stem cells to supply large amounts of angiogenic, anti-apoptotic and mitogenic factors [18,21], cell transplanted groups may have increased neoangiogenesis via a paracrine effect in the ischaemic myocardium. On the other hand, the culture medium injection group showed deleterious effects on angiogenesis, probably, due to an increased amount of various unfavorable cytokines such as TNFα and IL-6 that impair new vessel formation. It has been demonstrated that bone marrow cells strongly suppress T-lymphocyte proliferation [22,23]. In the present study, direct intramyocardial injection of SVF and BM-MNC to the ischemic myocardium substantially suppressed CD3 cell (T lymphocyte) and CD20 cell proliferation (Figure 5 and 6, respectively) and down regulated the production of inflammatory cytokines, such as TNFα, IL-6 and TIMP-1 (Figure 4; A, B and D respectively). These cytokines may be involved in the pathogenesis of heart failure or LV remodelling [24,25]. It has been previously shown that TNFα released from ischemic heart after acute MI, has been shown to reduce contractility, increases the production of other cytokines such as IL-1, IL-6 and TIMP-1, induces pathophysiological hypertrophy, promotes apoptosis of cardiomyocytes and other alterations of the extracellular matrix which finally accelerates LV remodeling [26]. In addition, serum levels as well as the local concentrations of inflammatory cytokines, especially, TNFα, are significantly increased in patients with chronic heart failure and these levels correlate with the degree of functional impairment [27,28]. Repeated TNFα infusion may lead to a permanent decrease in myocardial contractility [29]. An increasing number of experimental observations suggests that IL-6 is also capable of modulating cardiovascular function, exerting a negative inotrophic function. IL-6 can be expressed in myocardium under various forms of stress and, also, it has the ability to induce apoptosis, cardiac hypertrophy and fibrosis in myocardium [29]. Therefore, in the present experiment, IL-6 in the myocardium of the culture medium injected animals may have been upregulated by relative ischemia in the hypertrophied myocyte itself.

We focused on the role of MMP-1 activation for several reasons. It has previously been shown that BM mesenchymal stem cell transplantation reduces gene and protein expression of MMP-1 and TIMP-1, inhibits collagen deposition in the ischemic myocardium [30]. MMP-1 has been shown to play an important role in myocardial matrix degradation, which is associated with ischemic heart disease [31]. We observed that SVF transplantation inhibited gene expression of MMP-1 and TIMP-1, which might have influenced the collagen degradation in the myocardium. We noticed that severe fibrosis developed in the infarcted area in the control group with culture medium injection, whereas only limited fibrosis was seen in the groups receiving SVF and BM-MNC.

The results implicate the mechanisms and efficiency of using SVF as an alternative to BM in treating cardiac dysfunction. Our findings on the expression of inflammatory cytokines in the myocardium add another dimension to understanding the anti-inflammation role of SVF and BM-MNC transplantation in cardiac dysfunction. The potential anti-inflammation role of both SVF and BM-MNC transplantation is well recognized but needs to be further studied. It is obvious that the failed clinical trials [20,32] were carried out before we had sufficient understanding of how inflammation is involved and regulated following cell transplantation in heart disease.

## Conclusions

In conclusion, our data suggest that transplantation of SVF might be a useful therapeutic option for angiogenesis in chronic ischemic heart disease. Given the feasibility and the lower invasiveness required to obtain adipose tissues from patients, freshly isolated SVF could be widely used to treat patients with ischemic heart diseases along with other sources of stem cells such as BM-MNC. Although our study has provided data supporting the usefulness of SVF implantation into the ischemic myocardium, further studies are required to improve the reproducibility and to monitor long-term effects in larger animal models.

## Competing interests

The authors declare that they have no competing interests.

## Authors' contributions

GUP performed all the cell culture procedures, surgical procedures, histology and design of the manuscript. LPM participated in the animal studies. MF (Professor Masatoshi Fujita) performed critical review of the concepts, read and approved the final version. XL contributed to the histology and statistical analysis. EB participated in echocardiography. MF (Professor Michael Fu) participated in its design and coordination. All authors read and approved the final manuscript.
